# Dentistry and Gender Gap: An Overview of the Italian Situation

**DOI:** 10.3390/healthcare11060828

**Published:** 2023-03-11

**Authors:** Sara Bernardi, Maria Bruna Fulgenzi, Angela Rovera, Fabiola Rinaldi, Sara Trichilo, Serena Bianchi

**Affiliations:** 1 Department of Life, Health and Environmental Sciences, University of L’Aquila, 67100 L’Aquila, Italy; sara.bernardi@univaq.it (S.B.);; 2 Private Dental Practitioner, 67100 L’Aquila, Italy; 3 National Association of Italian Dentists (ANDI), 00153 Rome, Italy; 4 Dental Physical Sciences Unit, Centre for Oral Bioengineering, Institute of Dentistry, Queen Mary University of London, London E1 4NS, UK; 5 Department of Innovative Technologies in Medicine & Dentistry, Dental School, ‘G. D’Annunzio’ University of Chieti–Pescara, 66100 Chieti, Italy

**Keywords:** oral health, gender equity, dentistry

## Abstract

Recently, the issue of the gender gap in dentistry and in the leadership in the dental field has placed new challenges on dental profession representatives and politicians. Indeed, the inequity between genders in healthcare workforces represents a limit to the progression of those in the professions, inhibiting talented colleagues from accessing high positions in the academic world and not providing adequate role models to inspire future generations. The Italian population practicing dentistry in 2021 was composed of 74% males and 26% females. The aim of this study is to analyze the level of the gender gap in the dental field in Italy by focusing on the gender distribution of professional leaders in institutional category associations and invited speakers at dental conferences accredited for the Continue Education in Medicine program. The search strategy considered three different fields in the national dentistry scene: the dental committee boards, the union category associations, and the cultural field. The roles in the dental boards were retrieved from the website of the National Medical and Dental Committee from 2018 to 2022; the roles in the union category associations at the provincial, regional, and national levels were retrieved from the related web pages, considering the period from 2018 to 2021; and the information about the speakers at national conferences from 1 January 2019 to 31 December 2021 was retrieved from the National Agency for Regional Health Services (Agenzia nazionale per i servizi sanitari regionali (AGENAS)) websites. The extracted data were grouped and examined using descriptive statistics (frequency), and due to the categorical nature of the data, non-parametric tests (chi-square tests) were used to assess any differences between the two genders. The null hypothesis was that there were no statistical differences between the two genders’ distributions. A *p*-value was considered significant when <0.05. In addition, aiming to understand the representation of male and female dentists, a comparison of the distributions of males and females in the dental boards with the percentages of female and male dentists operating in Italy in 2021 was performed using a binomial test. The analysis of the data regarding the composition of the dental boards and of the union category association boards revealed a statistically significant difference in the distribution of the roles between the two genders at the regional and local levels, with a *p*-value of <0.05, and a significantly low representation of the women practicing dentistry (*p* < 0.05). In Italy, women practicing dentistry are underrepresented in dental organizations and in cultural events, given the low numbers of female dentists in leadership and/or speaker roles in the years 2018–2021. Even though the glass ceiling effect continues to affect Italian female dentists, initiatives and political measures have been taken to improve this condition and reach gender equity.

## 1. Introduction

The term “gender” indicates the identities of male, female, and gender-diverse populations in a social context. The World Health Organization’s (WHO) definition of gender refers to “the roles, behaviors, activities, attributes, and opportunities that any society considers appropriate for girls and boys, and women and men” [1].

Healthcare professions are traditionally distinguished by male prevalence, including the dental profession [2]. Even though the trend of women graduating in and practicing medicine and dentistry is increasing [3], the positions of directors and academic leaders still underrepresent this gender’s prevalence in the dental workforce.

Indeed, as reported by the Women Dentists Worldwide, which is part of the Federation Dentaire Internationale (FDI), over 60% of dentists in Europe are women [4]. In 2019, Spain reported that 65% of those who graduated in dentistry were female, and Germany reported similar values [3]. USA and Canada also reflected those numbers [5]. With regard to Italy, in 2021, it was estimated that 26% of the population practicing dentistry was female [6]. Only one study in 2014 reported differences in Italy regarding gender in the dental workforce, reporting how gender influences post-graduate education choices and the gender gap in the dental academic world [7].

Gender equity, however, does not reflect the gender distribution in the population actively working as dentists, as well as in the medical field [3].

The gender gap in the leadership in dental medicine is also a matter of health [8]; indeed, it represents a limit to the progression of the professionals, inhibiting talented colleagues from accessing higher positions in the academic world. In addition, the lack of adequate role models to inspire the future generation does not benefit the oral care programs for the general population [9].

Several programs aimed at improving diversity and inclusivity in health and healthcare professions are playing a crucial role in the awareness of the gender gap in the medical and dental categories. There is a problem, and not seeing it, or minimizing it, does not help. Indeed, a number of initiatives have been undertaken in different countries to address the gender gap.

In the UK, for example, the Royal College of Surgeons of England hosted Women in Surgery (WinS), an initiative aimed to empower and inspire women in their career progression and, therefore, to promote a gender balance [8].

In Italy, the category associations (the Italian National Dental Association (ANDI) and the Italian Association of Dentistry (AIO)) have, in the last few years, focused on the gender gap issue in dentistry. In particular, the ANDI has one of their delegates in the Working Group of Women in Dentistry of the Federation Dentaire International (FDI) [10] and has organized courses and conferences on gender in dentistry and on the role of dentists in the prevention and interception of domestic abuse [11]. The AIO organized the 2022 International Conference on the gender shift in dentistry, titling it “Focus on the gender shift in dentistry: A new perspective or a future challenge?” [12].

The company Dentsply Sirona has supported women’s empowerment in dentistry through (1) the introduction of an award (the Dentsply Sirona Smart Integration Award), (2) a program for female dentists to improve their skills as speakers and offer them the right opportunities (the Female Speaker Development Program), and (3) a forum where female dentists can network (the Dentsply Sirona Women) [13].

As stated by Piasecki et al., role models and appropriate mentorship inspire and guide the new generation, instilling self-confidence and pushing more females to put themselves in leadership positions. Cultural events, especially those accredited by Continue Medicine Education (CME) programs, are crucial for visibility and for role modeling opportunities [14]. The available literature confirms, however, an extensive gender imbalance in the invited speakers at dental conferences [14].

To our knowledge, an investigation on the gender differences in the positions of professional leadership (in dental board committees) and of speakers at events credited by the ICME program in Italy has not been performed.

The aim of this cross-sectional study is to analyze the level of the gender gap in the dental field in Italy by focusing on the gender distribution in professional leaders in institutional category associations and invited speakers at dental conferences accredited by the CME program.

## 2. Materials and Methods

### 2.1. Study Design and Description of the Objects of Investigation

The study design considered three different aspects of the national dentistry scene: the dental committee boards, the union category associations, and the cultural aspect.

The Italian system of medical and dental committees includes two distinct boards: the medical board (Albo Medici) and the dental board (Albo Odontoiatri). These boards represent a public authority established by Italian state law.

The medical and dental committees (in Italian, Ordine dei Medici e degli Odontoiatri) in Italy are distributed territorially for different provinces. There is also a national committee supervising and coordinating the local boards. The members of the boards are elected by medical and dental doctors.

The dental board roles are the president, the vice president, and counselors (which are variable in number). In addition, three or four members from the dental boards can be elected to be in the medical committee in different roles (such as vice president, treasurer, and counselor).

Regarding the category associations in Italy, the union category association with the highest number of subscriptions is the ANDI (Associazione Nazionale Dentisti Italiani), with boards at the provincial, regional, and national levels.

The provincial board members are elected by the members of the union. The provincial board members propose and elect the regional board members, and the regional board members elect the national board.

The cultural field is usually represented by events belonging to the CME program, and the details of each event and the speaker curriculum vitae are reported on the CME website of the National Agency for Regional Health Services (Agenzia nazionale per i servizi sanitari regionali (AGENAS)).

### 2.2. Institutional Roles in National and Local Dental Boards

The roles in the dental boards (named INSTITUTIONAL) of each province and in the national committee were retrieved from the website of the National Medical and Dental Committee (Federazione Nazionale Ordine dei Medici e degli Odontoiatri), considering the period from 2018 to 2022.

In particular, the following data were considered: gender, level (provincial or national), and position in the board.

### 2.3. Roles in ANDI

The roles in the ANDI at the provincial, regional, and national levels were retrieved from the related web pages, considering the period from 1 January 2019 to 1 January 2022 (UNION).

In particular, the following data were considered: gender, level (provincial or national), and position in the board.

### 2.4. CME Speakers

Regarding the cultural aspect (named CME), the national conference data from 1 January 2019 to 31 December 2021 were retrieved from the National Agency for Regional Health Services (Agenzia nazionale per i servizi sanitari regionali (AGENAS)) websites.

In particular, the following data were considered: gender, specialty, and the region where the event was organized. In cases with the same person, the event was counted once. Only graduated and professional dentists were included in this research.

### 2.5. Statistical Analysis

The extracted data were grouped and examined using descriptive statistics (frequency).

The three considered outcomes were as follows:

(1) Differences between the two genders’ distributions in the roles within the INSTITUTIONAL and UNION boards;
(2) The representation of male and female dentists in the INSTITUTIONAL and UNION boards;
(3) The influence of any discipline in the CME events on the gender distribution.

Regarding the first outcome, due to the categorical nature of the data, non-parametric tests (chi-square tests) were used to assess any differences between the two genders. The null hypothesis was that there were no statistical differences between the two genders’ distributions. A *p*-value was considered significant when <0.05. The calculations and graphs were made using GraphPad Prism 9 version 9.4.1.

Regarding the second outcome, a comparison of the distribution of males and females in local boards with the percentages of female and male dentists operating in Italy in 2021 [6] was performed using a binomial test. A *p*-value was considered significant when <0.05. The calculations and graphs were made using GraphPad Prism 9 version 9.4.1.

Regarding the third outcome, a two-way ANOVA was used to understand if any discipline influenced the gender distribution. A *p*-value was considered significant when <0.05. The calculations and graphs were made using GraphPad Prism 9 version 9.4.1.

## 3. Results

### 3.1. INSTITUTIONAL Roles in National and Local Dental Boards

**Figure 1:**
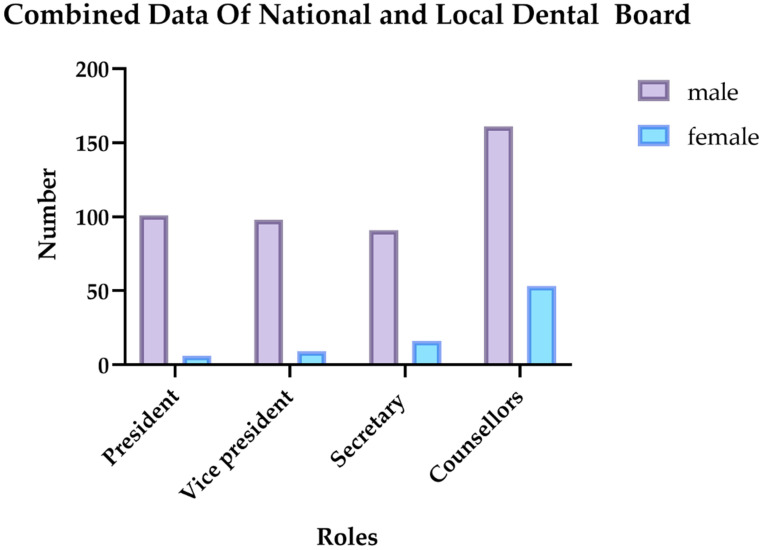
Graphical representation of the roles and gender distribution in national and local dental boards.

In Italy, in the period from 2018–2020, in total, the institutional dental boards were composed of 538 members, of which 454 were males and 84 were females, distributed across the different roles as shown in Figure 1.

As shown in Table 1, the figures associated with both national and local dental boards are higher for the male gender than for the female gender.

The difference between the distribution of the two genders in each role in national and local dental boards is statistically significant (Table 2).

**Figure 2:**
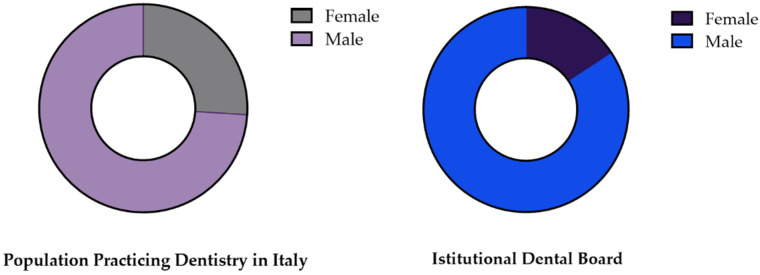
Graphical representation of the comparison of the composition of the combined data of national and local dental boards with that of the population practicing dentistry in Italy, represented, in terms of percentages, as 74% male and 26% female.

Regarding the representation of males and females, when comparing the percentages of the two genders in the population practicing dentistry and the percentages of the two genders in national and local dental boards (Figure 2), the binomial tests show a high significant discrepancy (Table 3).

### 3.2. UNION Roles in ANDI

**Figure 3:**
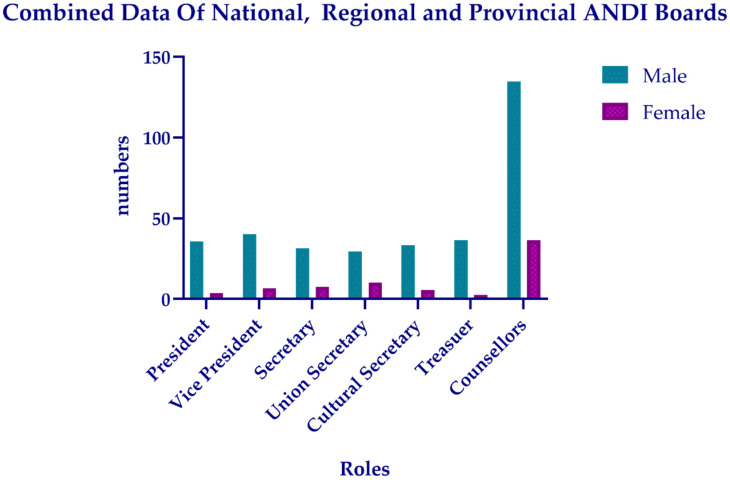
Graphical representation of the roles and gender distribution in national, regional, and provincial ANDI boards.

In Italy, in the period from 2018–2021, the total number of ANDI board members (at the national, regional, and provincial levels) was 1231, of which 1022 were males and 209 were females, distributed across the different roles as shown in Figure 3.

As shown in Table 4, the figures associated with all three ANDI boards are higher for the male gender than for the female gender.

The difference between the two genders is statistically significant for all the roles in the ANDI boards, combining the data of the national, regional, and provincial boards (Table 5).

**Figure 4:**
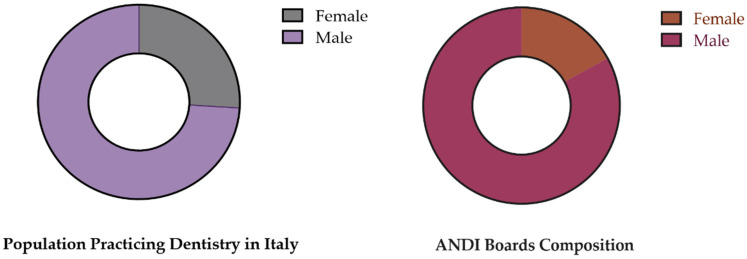
Graphical representation of the comparison of the composition of the combined data of national, regional, and provincial ANDI boards with that of the population practicing dentistry in Italy, represented, in terms of percentages, as 74% male and 26% female.

Regarding the representation of males and females in the ANDI boards (Figure 4), the binomial tests show a statistically significant difference (Table 6).

### 3.3. CME Speakers

Regarding the cultural events organized in the framework of the Continue Education in Medicine Program, from 1 January 2019 to 1 January 2022, the total number of dental speakers who were invited to hold conferences or seminars was 5428, of which 4613 were male and 815 were female.

As shown in Table 7, overall, the number of male speakers is significantly higher than that of female ones. Analyzing each discipline, pediatric dentistry and special dentistry do not present statistically significant differences. The specialty with more of a presence of males is implantology, while the specialty with more of a presence of females is orthodontics.

**Figure 5:**
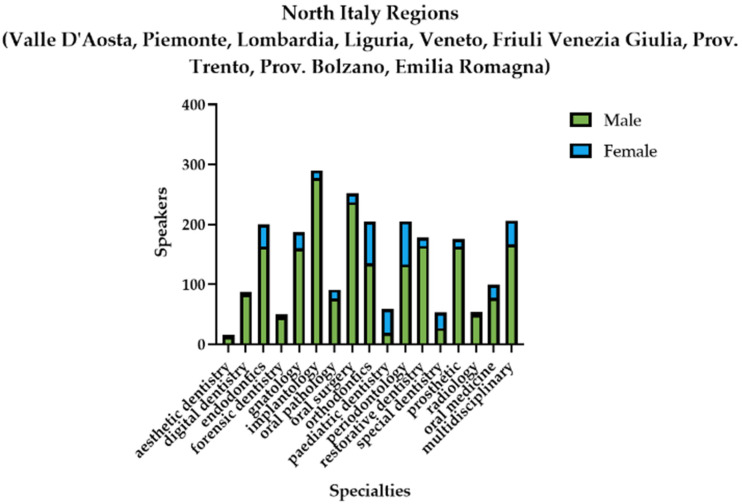
Graphical representation of the gender distribution of the speakers in the northern area of Italy.

**Figure 6:**
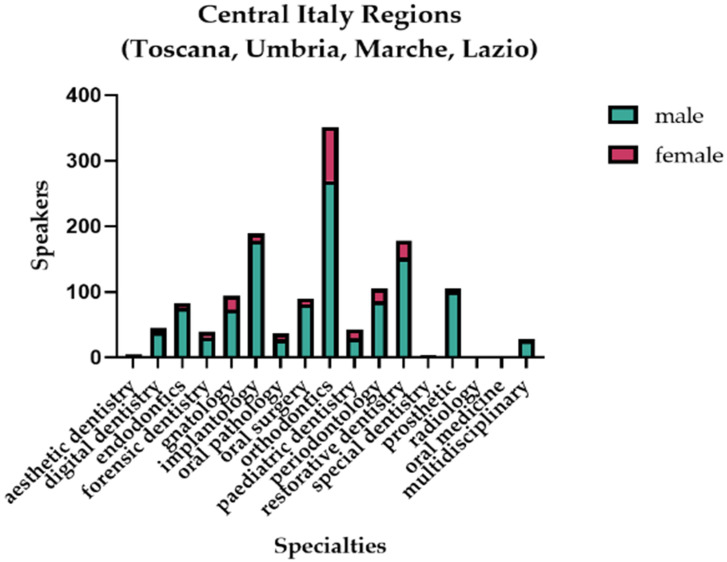
Graphical representation of the gender distribution of the speakers in the central area of Italy.

**Figure 7:**
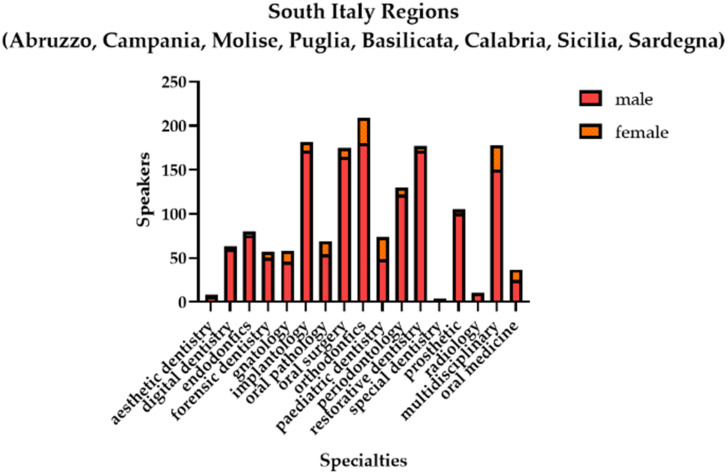
Graphical representation of the gender distribution of the speakers in the southern area of Italy.

In all of the three main areas of the country, the male presence was predominant, as shown in Figure 5, Figure 6 and Figure 7.

The difference in the presence of the two genders as speakers was statistically significant, in favor of the male gender in all three areas of the country (Table 8).

## 4. Discussion

### 4.1. 2018–2021: Dental Boards and Category Association: The Glass Ceiling Effect

Cerutti, in 2021, reported that 74% of the population of active dentists was male and 26% was female. The analysis and the comparison of the data regarding the compositions of the dental boards and the ANDI boards revealed not only a statistically significant difference in the distribution of the roles between the two genders at the regional and local levels (88% vs. 11% and 84% vs. 16%) but also a significantly low representation of the women practicing dentistry (*p* < 0.05).

These data agree with those presented in the literature about the leadership and representation of women in dentistry.

Gross et al. in their study about the “feminization” of dentistry in Germany, beyond analyzing how the union of the two Germanies after 1990 affected the demography of dentists, highlighted how German female dentists do not occupy large numbers of positions of leadership in private practices and in political positions, remaining underrepresented [15].

Indeed, Gross et al. reported that only 5% of female dentists practicing in Hamburg in 2010 actively participated in the politics of the dental chamber [15].

Our study confirmed these data and showed that 15% of women actively participate in institutional dental boards and that 11% of female dentists actively participate in politics (in the UNION category).

In Spain, the situation is even more severe: Ruiz et al. reported that the proportion of the female population among those practicing dentistry is about 50%, reaching the levels in Sweden and Norway [16]. However, according to their survey, Spanish female dentists consider themselves underrepresented in the general dental boards and note the presence of inequity between the two genders. This perception was confirmed by a later Ruiz et al. study [3] analyzing the distribution of leadership positions; regarding dental boards at the national level, females were represented at 15%, and at local levels, they were represented at 37%.

Even though the present study did not investigate the perception of female dentists regarding representativity and gender inequity, the data on representation are very similar to those reported in our study.

Indeed, female dentists are represented at 12% at the national level and 15% at the local level in institutional dental boards.

North America (Canada and the USA), instead, have not improved their situation: both the Canadian Dental Association and American Dental Association present ratios of leaders:dentists in the women category of 0.63 and of 0.72, respectively, suggesting the underrepresentation of female dentists [5].

Regarding the UK, the paper by O’ Brien et al. [17] on imbalanced dental boards moved the attention toward gender shift issues, and from 2020 to 2022, the boards increased the proportion of women to 40% and greater [18], which is, therefore, a higher percentage compared with the one found in our investigation.

Italian women dentists as well as those in other countries suffer the “ceiling glass effect”. Indeed, not only is most of the population practicing dentistry male, but also those women who might dedicate part of their careers to the political aspect of dentistry, such as dental associations or institutional boards, and taking on leadership positions, simply do not. As has been widely reported, this might be due to a lack of women practicing dentistry, who can also serve as role models for the future generation. Indeed, it is reported that when a woman takes on a leadership position in dentistry, she has less probability of being married or with children [3,4,5,19,20,21,22,23,24,25]. This scenario frightens new generations of young women who see themselves as forced to choose between a career or family, which is a scenario that young men do not perceive [26].

Indeed, in the examined countries, it is highlighted how the policies supporting families and relieving them of child care are still insufficient to properly aid women in coordinating the work at home and the work at dental practices.

All these factors contribute to not providing female dentists with potentially valid leaders and to favoring women’s underrepresentation.

### 4.2. Invited Speakers Have Been Men-Made

The findings from the analysis in the current study confirm the imbalance in the female presence at dental cultural events as invited speakers.

Indeed, the meetings organized in the framework of the CME, and accredited by the AGENAS, include a scientific committee or a scientific responsible that selects and invites the speakers.

It appears, therefore, that in educational meetings in the dental profession, there is a lack of awareness of trying to balance the gender gap, inviting mostly male speakers.

These results confirm what the literature in the medical fields affirms [27,28,29]: the panels of invited speakers at conferences show an imbalanced representation of the two genders.

A recent study by Koletsi et al. examined the panel of oral presentations and invited speakers in the meetings of the European Orthodontic Society from 2015 to 2020 [30]. Interestingly, they found that the panel of oral presentations was balanced between male and female speakers, but when they examined the panel of invited speakers, the results showed an imbalance between the two genders, being composed of predominantly males [30].

Our results show a significant difference between the two genders, with 23% of invited speakers being female vs. 77% of them being male at orthodontic CME events.

A similar study was performed examining U.K. dental conferences [8]. Only three conferences presented a balanced panel based on gender, raising the issue of the gender gap in the dental workforce [8].

In addition, a study by Psiaceki et al. assessed the presence of women as invited speakers in meetings and conferences organized by the main US prosthodontic societies [14]. The results of the study confirmed an imbalance and low presence of invited women speakers.

The gender imbalance in panels of speakers in prosthodontics was also present in our study.

Another study by Martorell et al. confirms this trend in Brazil [31]. The study by Martorell showed how the imbalance was different according to the considered discipline [31]. As in our study, pediatric dentistry showed no differences between the two genders.

Another interesting datum from our study is the one regarding the special dentistry discipline, the discipline regarding the oral care of people with special needs. Indeed, in this case, the difference between male and female speakers was also not significant.

In the study by Martorell et al., these types of specialties are attended more by women than by men, who, instead, appear more interested in oral surgery and prosthodontic disciplines [31].

A key factor the mentioned studies consider is the composition of the committees that should select the invited speakers. Psaceki et al. noted that scientific committees are usually male-composed and/or not sensitive to the gender gap issue [14].

Indeed, a “stereotype” that leads to a male predominance in invited speakers is that there are no women to invite [14]. Even though the male proportion of the population practicing dentistry might be slightly higher than the female one, our data and the data of studies available in the literature show that the proportions are not equal.

In addition, the lack of invited female speakers continues to perpetuate the lack of female leadership and role modeling, which, instead, could inspire young and capable women to actively participate in important positions and scientific activities.

Indeed, the study by Koletsi et al. showing no difference between the two genders in oral presentations demonstrates that there are women who are as dedicated to research and scientific activities as are men [30].

However, when the positions and authorship, as well as the editorial boards of scientific journals, are considered, the male gender continues to prevail.

Nkenke et al.’s analysis of the gender trend in oral and maxillofacial surgery scientific production showed an increase in female authorship from 1980 to 2010, but the proportions remained imbalanced, favoring the male gender [32].

More recently, Franco et al. highlighted how the COVID-19 pandemic decreased the proportion of women as corresponding authors in Central and South America [33].

Research authorship represents a fundamental requisite for career progression in academic dental fields.

Indeed, as reported by Lalloo, who assessed a lack of diversity in editorial teams, women who do not have role models as editors-in-chief at dental journals are not facilitated into participating in editorial boards [34], feeding the vicious cycle of the lack of progression in women’s careers in the academic field.

### 4.3. Future Perspectives

The considered years in the present study showed a scenario that has to be improved. However, the Italian government during the years 2018–2021 took a series of transversal and declinable measures in the dental profession to promote female empowerment, such as the following:

(1) The **National Recovery and Resilience Plan** (NRRP) has the aim of guaranteeing the same economic and social opportunities for men and women with reforms, education, and investments, with a view of gender mainstreaming by breaking down gender and generational discrimination. It fits into the path traced by the European Union Strategy and is built starting from a long-term vision [35]. The plan impacts dental professionals regarding female leadership and support in parenthood. In particular, NRRP includes the “Female enterprise fund” to support female entrepreneurship, impacting women in the third sector, such as private dental practices, and achieving more significant gender equality. The fund intends to financially support the creation of new women-owned businesses (of less than 12 months) and the development and consolidation of existing women-owned businesses (of more than 12 months). The expenses eligible for the fund are related to tangible fixed assets (machinery, plants, equipment, and building renovation work) for a maximum of 30% of the total eligible expense and intangible fixed assets (software, patents, licenses, and trademarks), cloud services (that are functional to the core processes of company management), employees (hired temporarily or permanently), working capital (raw materials, consumables, services, and rents), and leasing for a maximum of 20% of the total eligible expense (or 25% for enterprises of 36 months). The fund aims to finance 2400 companies by June 2026.
(2) The **Gender Equality Certification System** started in 2021. The aim of the project is the definition of a national gender equality certification system that accompanies and encourages the adoption of adequate policies to reduce the gender gap in all the most “critical” areas “(growth opportunities, equal pay for equal jobs, gender difference management policies, and maternity protection) [36]. The project is coordinated by the Department for Equal Opportunities in collaboration with the Ministry of Labor and Social Policies and the Ministry of Economic Development [36]. The measures of this project can affect private dental clinics in the improvement of the quality of the work of their female dentists, offering equal growth and formation opportunities.

Regarding, maternity protection and parenthood support, the Italian social security system dedicated to medical doctors and dentists (Ente Nazionale di Previdenza ed Assistenza dei Medici e degli Odontoiatri (Fondazione ENPAM)) in 2022 took an initiative “Baby Bonus” (in Italian Bonus Bebè) to give economical sustainment during the first months of maternity for female dentists who work as self-employed.

In detail, a subsidy equal to five twelfths to eighty percent based on income is given for five months (two before and three after birth).

In addition, an economical contribution of 2000 € is given per birth, aiming to help cover the expenses for babysitting services and maintenance.

(3) **Establishment of a Parliamentary Commission for women’s rights and gender equality with “guideline and control” tasks (4 November 2019).** The commission carries out investigations and monitoring activities on the presence of women in political-institutional life and in the world of public and private work in the country through the analysis of gender statistics elaborated by the responsible bodies, also promoting the balance between the professional and familial for women and men through the implementation of policies for the reconciliation between work and private life and the use of tools that encourage the sharing of family responsibilities [37]. This measure includes the possibility for new fathers to take “paternity leave” to allow the sharing of family responsibilities. In this case, dentists working in the public health system or working as employees can benefit from this measure to re-balance their working and career progression with family responsibilities.

In addition, the forecasts and the data on undergraduate dental students promise an increase in the number of women in the dental workforce as dentists, which could serve as the push for dental associations to promote gender equity in boards, research, and panels of speakers, providing role models and diversifying organizations, making them more efficient.

### 4.4. Strengths and Limitations

The limitations of this study were given by not considering the data from the AIO and the courses and cultural events taken online during the pandemic period. Furthermore, the data from the universities and institutions reporting female researchers and professors in dentistry have not been considered.

In addition, the age parameter was not considered. Indeed, if we consider the young generations, the trend in the population practicing dentistry goes toward a gender parity situation [12], aligning with other countries. Further investigations are necessary to compare how and/or if the gender gap is filled in different generations.

The strength of this study is the updated overview of the gender gap in dentistry, underling the underrepresentation of women at three different levels, showing that the gender issue is still present in Italy and unsolved.

## 5. Conclusions

The gender gap in dentistry is still an actual issue, and is unsolved in many countries, despite the attention given to the problem. In Italy, women practicing dentistry are underrepresented in dental organizations and at cultural events, given the low numbers of female dentists with leadership and/or speaker roles, in the years 2018–2021.

Even though the glass ceiling effect continues to affect Italian female dentists, initiatives and political measures have been taken to improve this condition and reach gender equity. Future studies are necessary to evaluate the effects of these political measures on filling the gap.

## Figures and Tables

**Table 1:** Roles in nationals and local dental boards.

Role in National Dental Boards	Male (*n*)	Female (*n*)	Role in Local Dental Boards	Male (*n*)	Female (*n*)
President	1	0	President	100	6
Vice President	1	0	Vice President	97	9
Secretary	1	0	Secretary	90	16
Counselors	4	1	Counselors	160	52

**Table 2:** Chi-square tests, dF (degree of freedom) values, and *p*-values to evaluate the different distributions of the genders in the different roles in national and local dental boards. dF = degree of freedom.

Role	Male (*n*)	Female (*n*)	Chi-Square Test	dF	*p*-Value
President	101	6	84.346	1	<0.05
Vice President	98	9	74.028	1	<0.05
Secretary	91	16	52.570	1	<0.05
Counselors	164	53	56.779	1	<0.05

**Table 3:** Binomial test for the comparison of the composition of the combined data of national and local dental boards with that of the population practicing dentistry in Italy. The discrepancy resulted as statistically significant.

Gender	Expected %	Observed %	95% CI of Observed %	Binomial Test	
Female	26.00	15.82	12.96 to 19.17	*p* (one-tailed)	<0.0001
Male	74.00	84.18	80.83 to 87.04	*p* (two-tailed)	<0.0001
TOTAL	100.0	100.00		Is discrepancy significant (*p* < 0.05)?	Yes

**Table 4:** Roles in national, regional, and provincial ANDI boards.

Role	Male (*n*)			Female (*n*)		
	National	Regional	Provincial	National	Regional	Provincial
President	1	19	87	0	2	9
Vice President	4	32	84	0	8	12
Secretary	0	17	77	1	4	18
Union Secretary	1	21	66	0	0	30
Cultural Secretary	1	19	80	0	2	15
Treasurer	1	21	87	0	0	8
Counselors	0	0	404	0	0	109

**Table 5:** Roles in the ANDI boards. Chi-square tests, dF (degree of freedom) values, and *p*-values.

Role	Male (*n*)	Female (*n*)	Chi-Square Test	dF	*p*-Value
President	107	11	78.102	1	<0.05
Vice President	120	20	71.429	1	<0.05
Secretary	94	23	43.085	1	<0.05
Treasurer	109	8	87.188	1	<0.05
Union Secretary	88	30	28.508	1	<0.05
Cultural Secretary	100	17	58.880	1	<0.05
Counselors	404	109	169.639	1	<0.05

**Table 6:** Binomial tests for the comparison of the composition of the combined data of national, regional, and provincial ANDI boards with that of the population practicing dentistry in Italy. The discrepancy resulted as statistically significant.

Gender	Expected %	Observed %	95% CI of Observed %	Binomial Test	
Female	26.00	16.98	14.98 to 19.18	*p* (one-tailed)	<0.0001
Male	74.00	83.02	80.82 to 85.02	*p* (two-tailed)	<0.0001
TOTAL	100.0	100.00		Is discrepancy significant (*p* < 0.05)?	Yes

**Table 7:** Numbers of male and female dental speakers at the cultural events registered in the framework of the CME program from 1 January 2019 to 1 January 2022. DF = degree of freedom. N.S. = not significant.

Disciplines	Male (*n*)	Female (*n*)	Chi-Square	DF	*p*-Value
16	4613	815	2657.480	1	<0.0001
**Type of Discipline**	**Male (*n*)**	**Female (*n*)**	**Chi-Square**	**DF**	***p*** **-Value**
Aesthetic Dentistry	24	5	12.448	1	<0.0004
Digital Dentistry	183	13	147.449	1	<0.0001
Endodontics	315	48	196.388	1	<0.0001
Forensic Dentistry	126	20	76.96	1	<0.0001
Gnathology	279	60	141.5	1	<0.0001
Implantology	627	34	532.0	1	<0.0001
Multidisciplinary	229	51	113.2	1	<0.0001
Oral Medicine	218	53	100.5	1	<0.0001
Oral Pathology	158	39	71.88	1	<0.0001
Oral Surgery	483	34	389.9	1	<0.0001
Orthodontics	584	181	212.3	1	<0.0001
Pediatric Dentistry	97	79	1.841	1	N.S.
Periodontology	341	99	133.1	1	<0.0001
Prosthetic	365	21	306.6	1	<0.0001
Radiology	63	5	49.47	1	<0.0001
Restorative Dentistry	488	45	368.2	1	<0.0001
Special Dentistry	33	28	0.4098	1	N.S.

**Table 8:** Two-way ANOVA of the gender distribution of the speakers in three areas of Italy. In all three areas, the variation in gender was statistically significant. * = level of significativity.

Two-Way ANOVA for Northern Region of Italy				
Source of variation	% of total variation	*p*-value	*p*-value summary	95% CI of difference
Specialties	31.06	0.3958	N.S.	52.90 to 131.8
Gender	41.79	0.0001	***	
**Two-Way ANOVA for Central Region of Italy**				
Source of variation	% of total variation	*p*-value	*p*-value summary	95% CI of difference
Specialties	54.56	0.0546	N.S.	26.47 to 86.12
Gender	22.73	0.001	**	
**Two-Way ANOVA for Southern Region of Italy**				
Source of variation	% of total variation	*p*-value	*p*-value summary	95% CI of difference
Specialties	32.53	0.3112	N.S.	44.05 to 105.5
Gender	42.14	<0.0001	****	

## Data Availability

The data will be available upon reasonable request from the corresponding author.
